# Emotional impact on Spanish health professional because of the COVID19 crisis

**DOI:** 10.1192/j.eurpsy.2022.676

**Published:** 2022-09-01

**Authors:** A. Alvarado Dafonte, L. Soldado Rodríguez, G. Ruiz Martinez

**Affiliations:** 1 Hospital Antequera, Usmc, Málaga, Spain; 2 Hospital Jaén, Usmc, Jáen, Spain

**Keywords:** Emotional impact, Health professional, COVID19

## Abstract

**Introduction:**

The COVID-19 pandemic has positioned health professionals around the world in an unprecedented situation, having to work in extreme conditions.

The reactions of healthcare personnel that concern us most are the negative psychological effects of the pandemic, such as exhaustion, moral injury, acute stress reactions, anxiety, depression or post- traumatic stress disorder.

**Objectives:**

To assess the impact of the COVID19 crisis on mental health of Spanish health professionals during the start of the pandemic.

**Methods:**

A descriptive, cross-sectional study is carried out, in which the population sample to be studied was the health professionals who responded to the online questionnaire designed to assess the emotional impact caused by the COVID-19 health crisis.

The questionnaire collects sociodemographic and labor data, which correspond to the independent variables of the study. The dependent variables correspond to the two scales used in the questionnaire (SAS and SASRQ scales)

**Results:**

The population sample was 473 people.

Analyzing the results of the SAS scale, 26.5% of the sample had anxiety symptoms in a normal range, 44.3% had mild-moderate anxiety levels, 24.4% marked-severe anxiety and 4, 9% had extreme anxiety levels.

The SARQ questionnaire assesses the presence of an acute stress disorder. In our study, 31.6% of those surveyed had this type of disorder.

**Conclusions:**

Immediate interventions and support for health professionals are essential to improve psychological resilience and avoid the appearance of mental health problems.

**Disclosure:**

No significant relationships.

